# Predicting smear negative pulmonary tuberculosis with classification trees and logistic regression: a cross-sectional study

**DOI:** 10.1186/1471-2458-6-43

**Published:** 2006-02-23

**Authors:** Fernanda Carvalho de Queiroz Mello, Luiz Gustavo do Valle Bastos, Sérgio Luiz Machado Soares, Valéria MC Rezende, Marcus Barreto Conde, Richard E Chaisson, Afrânio Lineu Kritski, Antonio Ruffino-Netto, Guilherme Loureiro Werneck

**Affiliations:** 1 Tuberculosis Research Unit, Clementino Fraga Filho Hospital, Federal University of Rio de Janeiro, Rio de Janeiro, Brazil; 2 Johns Hopkins University School of Medicine, Baltimore, Maryland, USA; 3 Department of Social Medicine, Faculty of Medicine of Ribeirão Preto, University of Sao Paulo, Brazil; 4 IMS/UERJ, Department of Preventive Medicine, NESC, Federal University of Rio de Janeiro, Rio de Janeiro, Brazil

## Abstract

**Background:**

Smear negative pulmonary tuberculosis (SNPT) accounts for 30% of pulmonary tuberculosis cases reported yearly in Brazil. This study aimed to develop a prediction model for SNPT for outpatients in areas with scarce resources.

**Methods:**

The study enrolled 551 patients with clinical-radiological suspicion of SNPT, in Rio de Janeiro, Brazil. The original data was divided into two equivalent samples for generation and validation of the prediction models. Symptoms, physical signs and chest X-rays were used for constructing logistic regression and classification and regression tree models. From the logistic regression, we generated a clinical and radiological prediction score. The area under the receiver operator characteristic curve, sensitivity, and specificity were used to evaluate the model's performance in both generation and validation samples.

**Results:**

It was possible to generate predictive models for SNPT with sensitivity ranging from 64% to 71% and specificity ranging from 58% to 76%.

**Conclusion:**

The results suggest that those models might be useful as screening tools for estimating the risk of SNPT, optimizing the utilization of more expensive tests, and avoiding costs of unnecessary anti-tuberculosis treatment. Those models might be cost-effective tools in a health care network with hierarchical distribution of scarce resources.

## Background

Tuberculosis is one of the most important health problems in the world, with more than 8 million new cases and almost 2 million deaths each year [1,2]. The detection and management of pulmonary tuberculosis (PT) is a principal aim of tuberculosis control programs. However, smear-negative pulmonary tuberculosis (SNPT) is an increasing clinical and epidemiological problem, particularly in areas that are affected by the dual tuberculosis/human immunodeficiency virus infection (TB/HIV) [3]. A recent DNA fingerprinting study from San Francisco attributed 17% of TB transmission in this low prevalence setting to patients with SNPT [4]. HIV infection has been associated with an increased incidence of SNPT [5] and a higher mortality rate among patients with SNPT [6]. In Brazil, almost 30% of PT cases among adults are SNPT [7,8].

Diagnosis of SNPT is a difficult task, and in developing countries, the majority of these cases has been treated only on the basis of clinical and chest radiographic findings. Without a standardized clinical work up, the misdiagnosis rates have been estimated as high as 35% to 52% [9-11]. Sputum cultures increase the sensitivity of diagnosis substantially, but at increased expenses and complexity. New diagnostic approaches that could identify patients with SNPT, such as nucleic acid amplification assays, are expensive and have not been validated in developing countries under field conditions [3]. Much effort has been employed in the development of predictive models for PT in developed nations, but the main focus has been to evaluate models for optimizing the use of expensive hospital respiratory isolation rooms for PT patients where AIDS cases are more prevalent [12-22].

The present study used both logistic regression and classification trees to develop a predictive model for SNPT among outpatients seen at the public health care system in Rio de Janeiro city.

## Methods

### Setting and patient selection

Adults with symptoms and/or signs suggestive of SNPT referred to the Chest Service of Clementino Fraga Filho University Hospital (HUCFF) at the Federal University of Rio de Janeiro, from April 1^st^, 1996 to December 30^th^, 1999, were invited to participate in this cross-sectional study. Patients, who reported more than 3 weeks of cough, and who had two consecutive samples of spontaneous sputum that were acid fast bacilli smear-negative, or who had an absence of expectorated sputum, were eligible. Patients receiving anti-TB treatment were not included. Patients were referred from seven community health care units (CHCU) and from the outpatient unit of HUCFF.

### Procedures

Patients underwent a standardized interview, with questions regarding demographic variables and clinical history (e.g.: smoking, alcohol abuse [23], HIV infection/AIDS [24]). A physical examination was performed and one chest physician using a standardized form evaluated chest radiographs. Evaluation of chest radiographs was not blinded to clinical predictors, but was for the results of TB cultures. Patients underwent sputum induction or bronchoscopy with bronchoalveolar lavage based on clinical criteria. All 551 patients underwent sputum induction, but bronchoscopy was performed in 331 patients (60%). Cultures were kept to assess Mycobacterium tuberculosis growth for 60 days. All patients were followed up for six months. When requested by the attending physician, HIV testing by ELISA was offered, with Western blot confirmation of reactive ELISAs. Patients who did not complete the established protocol or who were lost during clinical follow up were excluded. The Institutional Review Board of HUCFF granted approval for this study. Written informed consent was obtained from all study subjects.

### Radiographic analysis

Chest radiographs were classified as typical, compatible, atypical and normal. Typical were those considered as having any parenchymal infiltrate or cavity localized in the upper zone (defined as the area above the posterior third rib); compatible were those presenting a miliary pattern, pleural effusion or thoracic adenopathy, and atypical those showing any other abnormality.

### Bacteriology tests

All respiratory specimens were stained by the Ziehl-Neelsen method and cultured in Löwestein-Jensen media. Species identification was performed according to Kubica's method [25].

### Case definition

Confirmed SNPT cases were defined as those with a positive culture for *Mycobacterium tuberculosis *in respiratory specimen; presumptive SNPT as clinical improvement after three months of anti-TB treatment as judged by three different chest physicians in a blinded review. Non-PT was considered among patients whose acid-fast smears and culture for *M. tuberculosis *were negative and who had no chest radiographic changes after six months of follow-up.

### Statistical analysis

We used statistical softwares S-Plus 4.5 (Statsci. Seattle, WA) for constructing regression and classification trees and STATA 6.0 (Stata Corporation, College Station, TX) for performing multiple logistic regression.

First, we used both techniques to identify the most significant independent factors for SNPT diagnosis. Then the predicted probability of SNPT for each patient was estimated from each model, and compared to the actual outcomes. The area under the receiver operator characteristic (ROC) curve, sensitivity, and specificity were used to evaluate the performance of the models.

In logistic regression, backward elimination was used to select variables to be maintained in the final model, using 10% as criterion for statistical significance of associations. Associations between predictive factors and the outcome were expressed as odds ratios (OR) and respective ninety-five percent confidence interval (95%CI). We tested for two-way interactions, but none was significant (data not shown). We also generated a score system using the logistic regression coefficients, as follows: we divided all coefficients by the lowest one and rounded the estimated value to the next integer, as described elsewhere [26].

Classification and regression trees (or CART) build a binary classification system (tree) through recursive partitioning, so the data set is successfully split into increasingly homogenous subgroups. At each stage (node) the CART algorithm selects the explanatory variable and splitting value that gives the best discrimination between two outcome classes. A full CART algorithm adds nodes until they are homogenous or contains few observations (≥ 5 is the standard cut off in S-Plus). The problem of creating a useful tree is to find suitable guidelines to prune the tree. The general principle of pruning is that the tree of best size would have the lowest misclassification rate for an individual not included in the original data [27].

## Results

Between April 1^st^, 1996, and December 30^th^, 1998, 551 patients were eligible for enrollment. Five per cent of patients were excluded because of incomplete data regarding follow up and/or bacteriological results. Forty nine percent (270/551) of the patients were diagnosed as SNPT. Among the cases, 78% (210/270) were bacteriologically confirmed and 22% (60/270) were diagnosed based on clinical and radiological improvement with anti-TB drug therapy.

We divided the original sample with random allocation of patients in two equivalent groups, one for building the models and the other one for validating them. Table 1 shows the characteristics of study subjects. The mean age of patients with SNPT was 40 years old and 91% of those patients were ≤ 60 years old. No statistically significant association with SNPT was observed regarding sex, previous PT, close contact with active PT cases, diabetes mellitus, smoking, alcoholism, HIV infection or AIDS. Patients originating at primary health centers were more likely to have SNPT than those initially seen at the hospital, but this finding was not maintained in the logistic regression analysis.

Table 2 shows the variables significantly associated with SNPT diagnostics in logistic regression. Age ≤ 60 years, presence of weight loss (more than 10%) and typical chest radiograph were positively associated with SNPT, while the presence of spontaneous sputum was negatively associated with SNPT. Non-specific clinical symptoms such as fever, headache, pain in the face and throat pain were not significantly associated with SNPT diagnosis, as was not dyspnea, thoracic pain and sweatiness (data not shown). Neither the length of symptoms nor previous use of antibiotics was significantly predictive of SNPT (data not shown). The score system generated by using the logistic regression coefficients attributed two points for a typical X-ray, minus 1 point for the presence of sputum, one point for weight loss and two points for age < 60 years.

**Figure 1:**
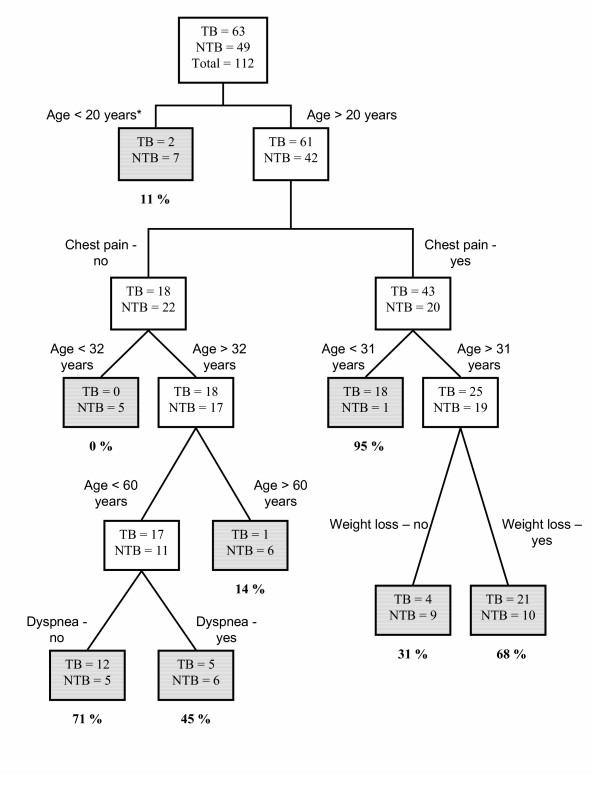
Classification tree for predicting smear negative pulmonary tuberculosis among patients with a typical chest radiograph.

**Figure 2:**
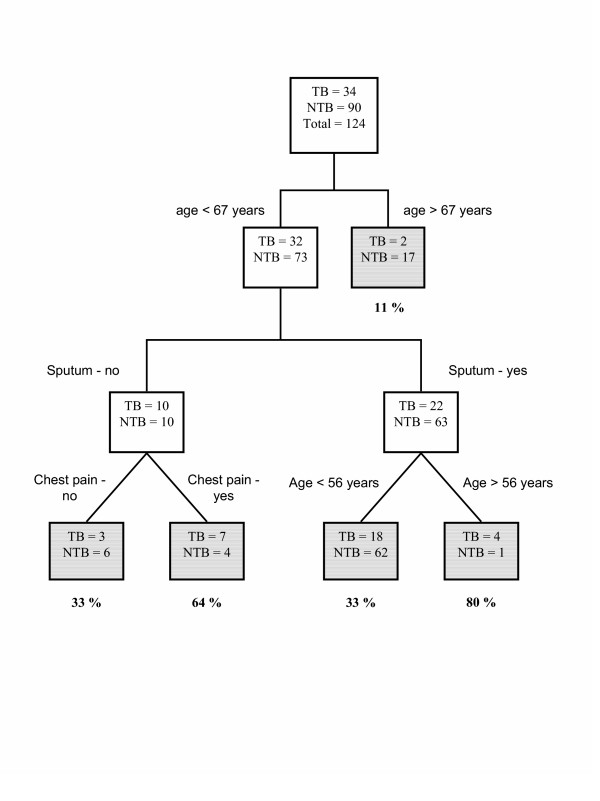
Classification tree for predicting smear negative pulmonary tuberculosis among patients with a atypical chest radiograph.

Figures 1 and 2 show the best classification trees, separated by the more powerful prediction variable: chest radiograph pattern. Therefore, two trees were constructed, simulating clinical practice. Other variables included using the classification and regression trees methodology, besides those identified by the logistic regression were chest pain, with positive prediction, and dyspnea, with negative prediction ability. As in logistic regression, age was also identified as an explanatory variable, and used for creating probabilities strata in both trees.

Table 3 shows the comparison of the predictive power of the different approaches in terms of the area under the ROC curve, sensitivity, and specificity. We looked for the cut off with best sensitivity and specificity relation (data not shown) for each model. Logistic regression and CART models had similar performance, considering both the original and the validation data sets.

## Discussion

The aim of this study was to evaluate the usefulness of logistic regression and CART models for the diagnosis of SNPT among outpatients in a high TB prevalence setting. Using clinical standardized interviews and chest radiographs it was possible to generate predictive models for SNPT with sensitivity ranging from 64% to 71% and specificity ranging from 58% to 76%. Evaluation of predictive models for PT respiratory isolation optimization has shown sensitivity ranging from 49% to 100%, and specificity from 46% to 86% [12-22]. It should be emphasized, however, that those models were applied to all PT suspect cases, including both smear negative and smear positive PT cases. Inclusion of smear positive cases potentially produces better results than obtained when analysis is restricted to smear negative patients. As smear negative patients present with a smaller mycobacterial burden, their clinical and radiographic findings may vary. Recently, a clinical radiological score for inpatients SNPT suspects was obtained through a retrospective chart review [28]. Its prediction ability was evaluated through likelihood ratios for each potential score and showed a potential utility if applied in settings with high prevalence of SNPT cases.

For SNPT, no single test provides sufficient accuracy for widespread use [3]. However, the techniques described here generated models for SNPT that were equivalent and were based on clinical variables and radiographic findings described elsewhere as useful for PT diagnosis [12,13,18-20]. In our study, the presence of expectoration was negatively associated with SNPT, as recently reported by Kanaya *et al*. [28].

The tree graphical probabilities results display or a score system may be easier to be performed under field conditions than the logistic regression formula, despite their equivalent performance. El-Sohl *et al*. [18] have already identified the utility of regression trees for PT diagnosis, with a slightly better result than logistic regression. The advantage of the models presented in this study is related to the variables identified in their development. They are readily available by conducting a routine clinical interview and by ordering a chest radiograph, so they could be applied in resource-limited settings. A possible example for using the score system, which might also be applied to CART models, using the probabilities of disease identified in our study, is the following:

• Score ≤0 – low probability of SNPT – indication of follow up at the primary health care unit, with observation and re-investigation over the next three months, time usually sufficient for clinical radiological evolution of markers of PT [29];

• Score from 1 to 4 – intermediary probability of SNPT – cases demanding further diagnostic investigation at a tertiary health care unit;

• Score ≥5 – high probability of SNPT – beginning empirical treatment for PT, with follow up at the primary health care unit.

Those models could also be useful as screenings tools, providing probabilities of PT, for evaluation of new diagnostics tests, as nucleic acid assays, for paucibacillary PT under clinical practice [30].

One limitation of the present evaluation was the absence of the tuberculin skin test. However, some authors had already described that in developing countries, tuberculin skin test is confounded by the high coverage of BCG vaccination, latent TB infection, the presence of nontuberculous mycobacteria, and anergy due to HIV infection or malnutrition, limiting its role in diagnostic algorithms [31]. Another limitation to be considered was the fact that only 56% of our patients underwent HIV testing; but the rate of TB/HIV co-infection in the present sample (21%) was close to the rate of TB/HIV co-infection rate in Rio de Janeiro's CHCUs at the same time (15%) [8].

## Conclusion

Predictive models should be validated in the populations where they will be applied. Based on the dynamic nature of biologic systems, any predicted outcome is vulnerable to change in response to new diseases, or a shift in the pattern of natural disease processes or progression. Such models must therefore be validated and optimized in each setting.

The current proposed models, if prospectively confirmed, might be useful in guiding health care workers in estimating the risk of SNPT, optimizing the use of more expensive tests, such as bronchoscopy and nucleic acid assays, and of unnecessary anti-PT treatment. The models could be useful as a cost-effective tool in a health care system with limited resources.

## List of abbreviations

SNPT Smear negative pulmonary tuberculosisPT pulmonary tuberculosis

TB Tuberculosis

HIV Human Immunodeficiency virus

HUCFF Clementino Fraga Filho University Hospital

CHCU Community health care units

ROC Receiver operator characteristic

CART Classification and regression tree

## Competing interests

The author(s) declare that they have no competing interests.

## Authors' contributions

FCQM coordinated the writing of the manuscript, was the principal investigator, had the concept, and design of the study, performed the data analysis, participated in data acquisition, LGVB drafted the paper, participated in data acquisition, SLMS drafted the paper, participated in data acquisition, VMCR drafted the paper, participated in data acquisition, MBC drafted the paper, participated in data acquisition, REC drafted the paper, ALK drafted the paper, was the principal investigator, and had the concept, and design of the study, ARN drafted the paper, helped design the study, performed the data analysis and GLW drafted the paper, helped design the study, performed the data analysis. All authors contributed to the interpretation of results, have read and approved the final manuscript.

## Pre-publication history

The pre-publication history for this paper can be accessed here:



## Figures and Tables

**Table 1:** Clinical and demographic characteristics of study subjects

	Pulmonary Tuberculosis		Non Pulmonary Tuberculosis				
Clinical and Demographic Characteristics*	N	%	N	%	OR	95%CI	p value
Age							
≤ 25	50	19.2	28	10.0	4.17	2.14–8.10	<0.01
26–60	186	71.5	195	69.9	2.23	1.32–3.74	<0.01
> 60	24	9.2	56	20.1	1.00	-	-
Sex							
Male	171	63.3	183	65.1	1.00	-	-
Female	99	36.7	98	34.9	1.08	0.76–1.53	0.66
Origin							
Primary Health Care Unit	192	71. 6	151	53.7	1.00	-	-
Tertiary Health Care Unit	76	28.4	130	46.3	0.46	0.32–0.66	<0.01
Previous PT							
Yes	65	24.3	78	27.8	0.84	0.57–1.23	0.36
No	202	75.7	203	72.3	1.00	-	-
Diabetes Mellitus							
Yes	18	6.9	20	7.2	0.96	0.49–1.85	0.89
No	243	93.1	258	92.8	1.00	-	-
Alcohol abuse							
Yes	51	19.0	62	22.1	0.83	0.55–1.25	0.37
No	217	81.0	218	77.9	1.00	-	-
Close Contact with PT							
Yes	121	45.2	112	40.0	1.24	0.88–1.73	0.22
No	147	54.9	168	60.0	1.00	-	-
Smoking							
Yes	114	42.2	152	54.1	0.62	0.44–0.87	<0.01
No	156	57.8	129	45.9	1.00	-	-
HIV +							
Yes	57	40.7	70	41.4	0.97	0.62–1.53	0.90
No	83	59.3	99	58.6	1.00	-	-
AIDS (Caracas Criteria)							
Yes	32	15.3	29	12.8	1.32	0.77–2.27	0.31
No	176	84.6	211	87.9	1.00	-	-

* The number of subjects may vary because of missing values

**Table 2:** Variables significantly associated with smear negative pulmonary tuberculosis (multiple logistic regression – final model)

	OR	95% CI	p value
Typical X-ray			
No	1.00		
Yes	2.63	1.55 – 4.45	<0.01
Presence of sputum			
No	1.00		
Yes	0.53	0.27 – 1.01	0.05
Weight loss			
No	1.00		
Yes	1.59	0.92 – 2.73	0.09
Age			
>60 years	1.00		
26–60	2.70	1.21 – 6.01	0.02
≤25	3.07	1.09 – 8.61	0.03

**Table 3:** Predictive performance of different multivariate models

	Sensitivity* (95% CI)	Specificity* (95% CI)	Area under ROC Curve* (95% CI)
Multiple logistic regression (original sample)	71.3 (62.4–79.1)	59.4 (50.7–67.7)	68.2 (61.9–74.6)
Multiple logistic regression (validation sample)	67.4 (58.6–75.4)	59.7 (50.9–68.1)	67.0 (60.6–73.4)
Multiple logistic regression using scores (original sample)	63.9 (54.8–72.4)	63.8 (55.2–71.8)	69.4 (62.8–76.0)
Multiple logistic regression using scores (validation sample)	65.9 (57.0–74.0)	60.5 (51.6–68.8)	68.1 (61.6–74.7)
CART (original sample)	63.7 (54.6–72.2)	75.5 (67.5–82.4)	74.9 (69.0–80.8)
CART (validation sample)	64.2 (55.6–72.2)	60.6 (51.9–68.8)	66.4 (60.0–72.8)

* Expressed in percents (%)
